# When Does Oxytocin Affect Human Memory Encoding? The Role of Social Context and Individual Attachment Style

**DOI:** 10.3389/fnhum.2018.00349

**Published:** 2018-09-20

**Authors:** Ullrich Wagner, Gerald Echterhoff

**Affiliations:** Department of Psychology, University of Münster, Münster, Germany

**Keywords:** oxytocin, memory encoding, social context, adult attachment style, joint action task

## Abstract

The neuropeptide oxytocin plays an essential role in regulating social behavior and has been implicated in a variety of human cognitive processes in the social domain, including memory processes. The present study investigates the influence of oxytocin on human memory encoding, taking into account social context and personality, which have previously been neglected as moderators for how oxytocin affects memory encoding. To examine the role of social context of encoding, we employed an established experimental paradigm in which participants perform a word-categorization task in either a joint (social) or individual (non-social) setting. To investigate the role of socially relevant personality factors, participants’ adult attachment style (AAS) was assessed. Previous research has identified attachment style as a potent moderator of oxytocin effects in the social-cognitive domain, but here we investigated for the first time its role in memory encoding. Participants were invited in pairs and received either placebo or oxytocin intranasally. Forty-five minutes later, they were instructed to react to different word categories within a list of successively presented words. This task was performed individually in the non-social condition and simultaneously with the partner in the social condition. After a 24-h delay, memory for all words was tested individually in a surprise recognition memory test. Oxytocin effects on memory accuracy depended on participants’ AAS. Specifically, oxytocin positively affected memory for participants who scored low on attachment dependence (who find dependence on others uncomfortable), but negatively affected memory for high scorers (who are comfortable depending on others). Oxytocin effects were not moderated by social vs. non-social context at encoding, and we discuss reasons for this outcome. Regardless of encoding condition or personality, oxytocin led to more liberal responding in the recognition memory test, which was also reflected in significantly higher false alarm rates (FARs) and a trend towards higher hit rates (HRs) compared to placebo. Overall, our results are consistent with an interactionist view on oxytocin effects on human cognitive functioning. Future research should further examine how oxytocin affects response biases via previous encoding and the ways in which biological dispositions linked to attachment style affect the process of memory encoding.

## Introduction

The neuropeptide oxytocin is predominantly known as the “social hormone.” In mammals, including humans, oxytocin is fundamentally involved in social relationships, specifically in mating and the bonding between mother and infant (for reviews, see Insel and Young, 2001; Ross and Young, 2009). In human research, these prosocial functions of oxytocin have inspired numerous experimental studies, which have examined how oxytocin helps guide social cognition and social decision making. Most of these studies have focused on economic decision making and on processes of emotion recognition, empathy and theory of mind (for overviews, see MacDonald and MacDonald, 2010; Bartz et al., 2011).

In contrast, only a small proportion of human oxytocin research has addressed memory formation (e.g., Ferrier et al., 1980; Heinrichs et al., 2004; Guastella et al., 2008; Di Simplicio et al., 2009; Rimmele et al., 2009; Herzmann et al., 2012; Liu et al., 2017). However, memory is a fundamental cognitive function that enables people to continually access relevant information and adjust their behavior accordingly after the encoding of experiences. In the social domain, memory allows individuals to maintain relevant information across different social encounters, and, hence, to adapt to anticipated social interactions in the future. Therefore, if oxytocin plays a significant role in human social cognition, it should also affect the encoding of new information.

Existing findings on oxytocin effects on human memory encoding are inconsistent. In early studies participants performed neutral verbal tasks using materials that did not consider social meaning. It was found that exogenous oxytocin had no or even negative effects on memory performance (Ferrier et al., 1980; Fehm-Wolfsdorf et al., 1984). In subsequent studies, researchers examined whether oxytocin would positively affect memory for socially relevant stimuli. In a study with verbal stimuli, Heinrichs et al. (2004) included a category of reproduction-related words associated with sex and baby care (e.g., “orgasm”, “pacifier”). Because oxytocin is relevant to basic social functions, the authors predicted that memory for such reproduction-related stimuli would be particularly sensitive to oxytocin administration. However, oxytocin was not found to enhance memory for this type of verbal stimuli. In fact, oxytocin even diminished memory performance in this study.

Overall, studies using verbal stimuli, such as those described above, are in the minority. Most of the studies on oxytocin effects on social memory have instead focused on memory for faces, which represent a natural type of stimulus relevant to social perception and interpersonal relationships (Tsao and Livingstone, 2008; Little et al., 2011; Simion and Giorgio, 2015). However, the findings on facial stimuli are inconsistent as well. For instance, memory-enhancing effects have been reported for faces regardless of emotion expression (Rimmele et al., 2009), only for faces with a positive expression (Guastella et al., 2008), or only for faces with neutral or negative expressions (Savaskan et al., 2008). In still other studies, there was no evidence for memory-enhancing oxytocin effects for faces at all (Di Simplicio et al., 2009; Herzmann et al., 2012; Bate et al., 2015). For example, Di Simplicio et al. (2009) report no effect on actual memory performance, but only on emotional classifications of facial stimuli. Likewise, Bate et al. (2015) found that oxytocin did not affect memory accuracy *per se* in a recognition memory test for faces, but rather induced more liberal responding, i.e., an enhanced general willingness to accept any stimulus in the memory test as previously seen.

Recent theorizing about how oxytocin affects human social cognition and behavior (Bartz et al., 2011) suggests that existing findings are inconsistent at least partly because oxytocin’s effects are sensitive to context and person factors. That is, oxytocin might not exert general effects that are observable across all situational conditions and across all subgroups of a population. Rather, the extent and direction of oxytocin’s effects on social cognition and behavior may actually be determined by context and personality factors (Bartz et al., 2011, 2015; Olff et al., 2013; Di Simplicio and Harmer, 2016). Indeed, an extensive review of the literature led Bartz et al. (2011) to conclude that oxytocin effects are typically moderated by situational and/or individual-difference factors. These authors thus propose an interactionist approach, which shifts the focus from the question of whether oxytocin affects a cognitive process *per se* to the more differentiated question of when and for whom it has such effects.

With regard to the inconsistent findings on oxytocin and human memory reported above, this interactionist approach suggests that simply defining social meaning by the stimulus material to be learned (e.g., facial vs. non-facial stimuli) may possibly fall short of targeting the most potent social determinants on which the effects of oxytocin on memory depend, i.e., social context and social personality. Therefore, in the present study, we investigate the role of social context and social personality as stimulus-independent moderators of oxytocin effects on memory formation.

Regarding social context, we employed an established distributed task-encoding paradigm that manipulates the social nature of the encoding situation (Eskenazi et al., 2013; Wagner et al., 2017). Specifically, in this paradigm participants perform a word categorization task either alone (non-social context) or simultaneously with another study participant (social context), where the two participants have to attend to different word categories. Previous research has demonstrated memory-enhancing effects due to the manipulation of social vs. non-social nature of the encoding context with this paradigm. Specifically, a surprise memory test performed by each participant individually after the word categorization task revealed enhanced memory performance for words from the partner-relevant word category presented in the social context condition (Eskenazi et al., 2013; Wagner et al., 2017). We assume that oxytocin would increase this specific memory-promoting effect of social context.

There are two major advantages to defining the encoding situation as social vs. non-social in this context-related way, compared to previous approaches that have relied on the social meaning of the presented stimuli themselves (Rimmele et al., 2009; Herzmann et al., 2012). First, defining social context by acting simultaneously together with a real person vs. acting alone is a more ecologically valid conceptualization of sociality than just presenting a static picture of a face of another person, because the mere viewing of a face might not reliably create a setting that participants perceive as really socially relevant (Risko et al., 2012; Schilbach et al., 2013). Second, when a real interaction partner defines the sociality of the situation, the same stimulus can be experimentally assigned to a social or non-social encoding condition. As a consequence, this approach does not suffer from possible confounds of stimulus-based manipulations of sociality, which can hamper the interpretation of effects. For example, facial stimuli are not only socially more relevant than other stimulus classes, but they are also visually more complex and encountered more frequently than most other stimulus types in everyday life (Goldstein and Chance, 1971; Diamond and Carey, 1986). These confounding factors might explain differences between conditions found in previous studies.

Apart from social context, the other stimulus-independent moderator that we aimed to address in the present study according to the interactionist approach was social personality. More specifically, we assumed that an individual’s attachment style would moderate oxytocin’s effects on memory. We specifically assumed attachment style as a potentially critical interindividual factor because attachment style, more than other personality characteristics, is directly linked to the basic biological functions of social attachment and bonding in which oxytocin is predominantly involved (Insel and Young, 2001; Ross and Young, 2009). In humans, attachment style has been linked to individual oxytocin levels and to genetic oxytocin receptor polymorphisms, so that it appears to reflect an endophenotype determining the responsiveness of an individual’s oxytocin system (Bartz et al., 2015; Shamay-Tsoory and Abu-Akel, 2016). Consistent with this view, attachment style has already been identified as a potent moderator of oxytocin effects on several types of human cognition and social behavior in previous studies (Bartz et al., 2010b, 2015; Olff et al., 2013; Fang et al., 2014; Waller et al., 2015). Hence, we assumed that such moderating influence of attachment style would likewise be observed in the case of memory of encoding.

Because we were specifically interested in oxytocin effects on the process of memory encoding (initial storage of new information), but not memory retrieval (getting access to previously encoded information), we made sure that oxytocin was pharmacologically active only at encoding. For this purpose, pharmacological treatment was administered before encoding, while retrieval (memory testing) took place on the next day, after oxytocin washout. Most of the previous studies cited above likewise administered oxytocin before stimulus encoding, but retrieval testing mostly took place immediately thereafter. Thus, oxytocin could have affected both encoding and retrieval in those studies (for exceptions, see Guastella et al., 2008; Rimmele et al., 2009; Herzmann et al., 2012). This unclear attribution of effects may have contributed to the inconsistency of the results across studies.

Taken together, the primary goal of the present study was to apply, for the first time, the interactionist approach to study how key social moderators influence oxytocin’s effects on memory encoding. Specifically, we hypothesized that social context and attachment style determine the individual perception of the social nature of the situation at encoding. This perception should influence downstream effects of oxytocin-dependent encoding processes. The interactionist approach and research on the social functions of oxytocin suggest that these variables (social context and attachment style) are likely to influence the socially driven salience of perceived stimuli, which is regarded as a basic mechanism for how oxytocin affects human cognition (e.g., Bartz et al., 2011; Shamay-Tsoory and Abu-Akel, 2016). According to this account, oxytocin, in concert with a dopaminergically driven attention mechanism, determines the orienting towards stimuli that are perceived as socially salient (Shamay-Tsoory and Abu-Akel, 2016). Because the perception of social salience is most likely also influenced by context conditions and by individual predispositions of attachment style, these factors should affect the extent and direction of these oxytocin-driven salience effects. Regarding context, we hypothesize that oxytocin should exert a positive effect specifically in the social, as opposed to the non-social context, on memory encoding of partner-relevant words, thus increasing the previously demonstrated joint encoding effect (Eskenazi et al., 2013; Wagner et al., 2017). Regarding attachment style, we expected, in line with most pertinent previous studies, differential effects for individuals displaying a personality of low vs. high social orientation in their dispositional attachment style, with more beneficial effects of oxytocin in the former group (Bartz et al., 2010a, 2011, 2015; Olff et al., 2013). However, the previous studies were not consistent with regard to which specific aspect of attachment style moderates oxytocin effects, and we had no a priori prediction on the differential pertinence of different aspects of attachment style to memory encoding. We therefore formulated no differential hypotheses for the different facets of attachment style (dependence, closeness, anxiety; see Collins and Read, 1990).

Apart from these expected moderations by social context and social personality, overall effects of oxytocin on either memory *per se* (e.g., Rimmele et al., 2009) or response bias in memory (e.g., Bate et al., 2015) might also occur. However, given the inconsistent evidence of general oxytocin effects on memory, we did not formulate any specific predictions for such main effects of oxytocin treatment.

## Materials and Methods

### Participants

Forty-eight university students (mean age = 24.8 years, *SD* = 4.6 years) were assigned to receive either oxytocin or placebo in a double-blind fashion. Pharmacological treatment represented a between-subjects factor, while social context was manipulated as the within-subjects factor, as described below. Exclusion criteria were self-reported allergic reactions towards parabens (an ingredient of the nasal spray), current illness or medication, or previous psychiatric or neurological diseases. Furthermore, in line with the majority of human oxytocin studies, women were excluded from participation because of the risk of uterine contractions (MacDonald and MacDonald, 2010; MacDonald et al., 2011).

Our sample size corresponds to (or even exceeds) sample sizes of relevant previous studies that have reported significant oxytocin effects (with effect sizes of a usually upper medium or high magnitude) in designs where oxytocin vs. placebo treatment was manipulated between participants (e.g., Guastella et al., 2008; Di Simplicio et al., 2009; Rimmele et al., 2009). A formal power analysis using G*Power (Faul et al., 2007), assuming an effect size of at least $\eta_{p}^{2}$ = 0.10 (upper medium magnitude) in a mixed analysis of variance (ANOVA; including 2 × 3 repeated measures in addition to the dichotomous between-subjects factor, as in the present case), confirmed that with an alpha level of 0.05 and test power of 0.80, an overall sample of 44 participants is sufficient to detect such effects in the between-subjects comparison as well as interactions of the between-subjects and within-subjects factors. We increased this sample size by about 10%, allowing for possible drop-out of some participants. Indeed, one participant had to be excluded from statistical analysis because of extremely low memory performance that did not exceed guessing level. Thus, the final sample encompassed *n* = 23 participants in the placebo condition and *n* = 24 participants in the oxytocin condition.

Participants were recruited individually, but pairs of individuals were invited to perform the tasks on the same date. Post-experimental questioning indicated that none of the pairs consisted of two persons who were already well acquainted with each other. The study was carried out in accordance with the recommendations of the local ethics committee at the University of Münster with written informed consent from all subjects. All subjects gave written informed consent in accordance with the Declaration of Helsinki. The protocol was approved by the ethics committee at the Department of Psychology at the University of Münster.

### Procedure and Task

Upon arrival, the two participants in each pair had the opportunity to get to know each other in a first personal encounter. They were introduced using their first names and seated next to each other to sign the informed consent. A few minutes later, they were led into separate rooms, where each of them self-applied a nasal spray that either contained oxytocin (24 I.U.) or placebo (composed of the same solution without oxytocin). Intranasally administered neuropeptides become maximally available in the central nervous system after a delay of about 45 min (Born et al., 2002); during this interval, participants were kept busy doing an unrelated non-verbal filler task comprising different aspects of face processing (Radke et al., 2013; Fang et al., 2014).

Then, after the 45-min delay, they performed a word categorization task, in which they (incidentally) encoded verbal material under social vs. non-social encoding conditions (Eskenazi et al., 2013; Wagner et al., 2017). Specifically, each participant performed two blocks of this word categorization task: one individual-task block in which they worked alone (non-social context condition) and a joint-task block in which they worked simultaneously with the other participant (social context condition), where each participant in the pair was assigned a different word category (see details below).

**Figure 1:**
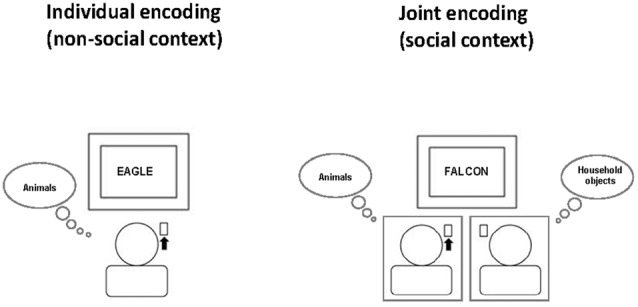
Social context manipulation at encoding. Participants always had the task to press a key whenever a word of a specific semantic category (e.g., animals) appeared on the screen. They performed the word categorization task once individually (non-social context, left) and once jointly with another participant (social context, right) who at the same time had the task to press a key in response to a different semantic category (e.g., household objects). The order of social and non-social context conditions was balanced across subjects. Participant were told that during individual task performance, the other participant would perform an unrelated other task in the adjacent room. This example illustrates the non-social and social context condition for the person on the left who has the task to respond to the word category “animals”. The black arrow indicates that this person has to press the key for the currently presented word.

For the word categorization task, subjects first read the task instructions. Then, each participant was assigned one of three word categories (animals, fruits/vegetables, household objects) and was instructed to respond as fast and as accurately as possible by pressing a specified key whenever a word of this assigned category appeared on the computer monitor and to do nothing whenever a word from another category appeared (Go/NoGo task). The two participants of a pair were always assigned to different word categories. For example, one participant always had to respond to animals (but not to household objects or fruits/vegetables), while the other participant always had to respond to household objects (but not to animals or fruits/vegetables). Each participant performed this task once alone (non-social condition) and once simultaneously with the partner in the pair (social condition), with the same category assignment across the two conditions (see Figure 1, for illustration). During individual task performance, each participant was led to believe that the other person would perform another independent task unrelated to the word categorization task. Thus, from the participant’s perspective, there were three word categories according to task assignment: “Self” (words to which oneself always had to respond), “Other” (words to which oneself never responded, but the partner did during joint task performance) and “None” (words that never required any response from either partner).

The word material consisted of 216 German nouns (72 words denoting animals, 72 words denoting fruits/vegetables and 72 words denoting household objects), subdivided into three parallel sets of words (each containing 24 nouns from each category). Two of these sets were used at encoding in the joint and individual task conditions; the third set provided the lures for the recognition memory test performed at retrieval testing on the next day (see below). The word sets were matched between and within each of the three word categories for word length and word frequency. Assignment of word sets to the joint and individual task conditions was balanced across subjects. To reduce potential biasing effects of single words within the lists, we also semantically matched words within in each category. For example, a bird in the word category “animals” in one word set was paralleled with another bird in the other word sets (e.g., falcon, eagle, vulture). The categorization task was implemented by E-Prime 2 software. Participants responded with one of two keys on the computer keyboard, one assigned to each participant of a pair. Stimuli were presented in random order. Each trial started with a fixation cross of 500 ms, followed by a stimulus word presented for either 1,500 ms or 3,000 ms. (This factor of stimulus duration was only exploratory and served to vary overall levels of memory performance. This exploratory factor did not influence any oxytocin effects and is therefore not further considered here).

Task instructions were the same during individual and joint conditions, i.e., to respond as fast and as accurately as possible to words from the “Self” category (which always remained the same for both individual and joint task performance). Thus, any performance differences between joint and individual conditions can be attributed to involuntary influences of the awareness that another participant was simultaneously involved in the task during joint task performance (with a different word category assignment). The order of the individual vs. joint condition of task performance, as well as the assignment of specific word categories to “Self”, “Other” and “None” conditions were counterbalanced across subjects. In the individual-task condition, the word categories “Other” and “None” were equivalent, because the partner was not involved. Still, as in previous studies, the same three word category labels as in the respective joint task performance conditions were used here, so that each of the three word categories “Self”, “Other” and “None” in the joint task condition had words from the same semantic category as a corresponding control in the individual task condition.

Both the individual and the joint task block started with a short practice run, using additional words not included in the word lists for the main run. We attempted to keep all situational factors other than the social nature of encoding as similar as possible between individual and joint task conditions. Thus, participants remained physically separated in both conditions, performing the task at different computers in different rooms. Physical separation ruled out confounds with the presence (vs. absence) of perceptual cues generated by the partner in the social condition (see Wagner et al., 2017). The two participants in a pair performed the task together on one computer only in the initial practice run of the joint task block. This was done to promote the perception of the social-interactive nature of the task in this condition, as opposed to the individual condition. Participants were then separated to perform the critical joint task trials, so that the only actual difference to the individual condition was the knowledge that now the other person was simultaneously involved, while confounding influences of actual perceptual cues generated by the partner were ruled out.

As in our previous study (Wagner et al., 2017), we took measures to ensure that the involvement of the partner could not be simply “forgotten” during joint task performance given physical separation. To this end, the program was individualized and included a feedback screen after each word presentation, indicating which would have been the correct response in this trial, i.e., a keypress by oneself, a keypress by the partner, or no keypress. This feedback was given in a personalized manner by using the first names of the participants and their partners. That is, at the end of each single trial, the feedback “keypress [first name of participant]”, “keypress [first name of participant’s partner]” or “no keypress” appeared on the screen. (Note that this feedback did not contain information about the partner’s actual performance, but only repeated the assignment of word categories that was already known from the general instructions.) This kind of feedback was also given in the individual task condition, where only the two feedback alternatives “keypress [first name of participant]” or “no keypress” were applicable.

After completing the individual and joint task blocks, participants left the laboratory. They returned individually after a 24-h delay to perform the memory retrieval session. This delay served to exclude effects of pharmacological treatment on retrieval functions. To reduce the likelihood of intentional rehearsal during this time delay, we phrased the instructions at encoding on day 1 without mentioning the upcoming memory test. Participants were merely told that some additional tasks would be performed on the next day.

At retrieval on day 2, a surprise recognition memory test was performed for all words that had been presented during the previous word categorization task. In this test, 216 words altogether were presented successively on the computer screen in random order. Out of these, 144 were words that participants had read during the categorization task and 72 were new. Participants had to indicate for each word whether it was “old” (presented during the categorization task) or “new”. For “old” answers, participants also indicated whether their answer was based on an experience of “remembering”, that is, recollection of the word as a vivid memory accompanied by details of the encoding episode, or of “knowing”, that is, a familiarity-based memory without retrieval of episodic details (Tulving, 1985; Yonelinas, 2002). This allowed us to additionally explore, for items indicated as “old”, the possibility of differential shifts in the two types of access to episodic memory that might be induced by oxytocin (Guastella et al., 2008; Rimmele et al., 2009; Liu et al., 2017).

As in the previous studies from Eskenazi et al. (2013) and Wagner et al. (2017), we also first presented a free recall test before the recognition test. However, as could be expected due to the extended retention interval of 24 h, overall recall performance was very poor (at floor level for many participants) and thus too low to allow statistical analyses. Results in the present study are therefore based only on analyses of recognition memory performance.

Finally, we assessed Adult Attachment Style (AAS, Collins and Read, 1990) as a social personality factor relevant to oxytocin effects (Bartz et al., 2010b, 2015; Olff et al., 2013; Waller et al., 2015). The questionnaire distinguishes three different facets of individual attachment style, i.e., Dependence, Closeness and Anxiety, which we considered as possible moderators of oxytocin effects.

As a control for possible unspecific mood effects of oxytocin, participants filled out the Positive and Negative Affect Schedule (PANAS; Watson et al., 1988) immediately before and after encoding at day 1 and immediately before and after retrieval testing at day 2.

## Results

The alpha level in all analyses was set to 0.05 (two-tailed). We first ran a 2 Pharmacological Treatment (oxytocin vs. placebo) × 2 Social Context (joint vs. individual) × 3 Word Category (self vs. other vs. none) mixed ANOVA, with pharmacological treatment as a between-subjects factor and recognition memory performance (regardless of the Remember/Know distinction for “old” answers) as the dependent measure. Memory accuracy, the primary measure of memory performance, was calculated according to standard procedures of recognition memory analysis (Snodgrass and Corwin, 1988) as the parameter P_r_, i.e., the difference between hit rate (HR, i.e., the proportion of old items correctly classified as old) and false alarm rate (FAR, i.e., the proportion of new items falsely classified as old). In contrast to raw values of HR and FAR, this parameter represents an indicator of genuine memory accuracy because it is not affected by the individual response bias in a recognition test, i.e., the extent to which a participant, when in doubt, generally tends to accept an item in the recognition test as an old item.

Results on P_r_ obtained after oxytocin vs. placebo treatment are shown in Table 1, overall and separately for the different experimental encoding conditions. There was only a strong main effect of word category, indicating better memory for words from the “self” category than for words from the other categories (*F*_(2,90)_ = 22.49, *p* < 0.001; P_r_ Self = 0.45, P_r_ Other = 0.30, P_r_ None = 0.28). This reflects the well-known “self-reference effect” in memory (Symons and Johnson, 1997) that was likewise observed in the data from Eskenazi et al. (2013) and Wagner et al. (2017). There were no other statistically significant ANOVA effects, including all effects that contained the factor Pharmacological Treatment (all *F*s < 1.24, *p*s > 0.27).

**Table 1:** Mean bias-corrected recognition memory accuracy (P_r_ = HR − FAR, with *SD*s in parentheses) as a function of pharmacological treatment and social context condition at encoding (joint vs. individual) for the three word categories.

	Word category	Placebo	Oxytocin
Joint Encoding	Self	0.475 (0.204)	0.422 (0.141)
(Social Context)	Other	0.342 (0.209)	0.292 (0.157)
	None	0.272 (0.161)	0.273 (0.193)
Individual Encoding	Self	0.467 (0.184)	0.420 (0.174)
(Non-social Context)	Other	0.290 (0.191)	0.292 (0.131)
	None	0.268 (0.216)	0.302 (0.186)
Total		0.349 (0.148)	0.331 (0.112)

We also performed analogous analyses separately for HR and FAR. There was a significant main effect of pharmacological treatment for FAR and a statistical trend towards a main effect of pharmacological treatment for HR, both resulting from higher values for both parameters after oxytocin than after placebo (FAR: Placebo, 0.26; Oxytocin, 0.37; *F*_(1,45)_ = 5.131, *p* = 0.028, $\eta_{p}^{2}$ = 0.102; HR: Placebo, 0.61; Oxytocin, 0.69; *F*_(1,45)_ = 2.89, *p* = 0.096, $\eta_{p}^{2}$ = 0.060). For HR, there was also a highly significant effect of word category (*F*_(2,90)_ = 34.48, *p* < 0.001; HR Self = 0.76, HR Other = 0.61, HR None = 0.59), paralleling the respective effect found for P_r_. No other effects were significant for either HR of FAR (all *F*s < 1.14, *p*s > 0.32).

**Figure 2:**
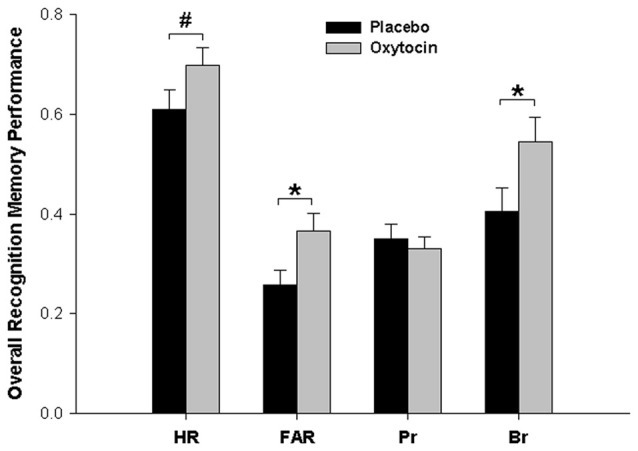
Main effects of oxytocin vs. placebo treatment on different recognition memory parameters. Compared to placebo, oxytocin led to more liberal responding at memory testing (increase in the response bias parameter B_r_), as reflected also in a higher false alarm rate (FAR) and, as a trend, a higher hit rate (HR). In contrast, there was no overall effect of oxytocin on actual bias-corrected memory accuracy (P_r_). *p < 0.05, ^#^*p* < 0.10.

In view of the parallel shifts in both hits and false alarms, we also directly calculated the response bias parameter B_r_ = FAR/(1 − P_r_) for each participant (Snodgrass and Corwin, 1988) to test whether this pattern was due to a general change of the response bias induced by oxytocin treatment (values of B_r_ > 0.5 indicate a liberal response bias, i.e., a tendency to decide for “old” in cases of uncertainty, while values of B_r_ < 0.5 indicate a conservative response bias, i.e., a tendency to decide for “new” in cases of uncertainty). This was actually the case, as overall B_r_ was higher after oxytocin than after placebo (Placebo, 0.40; Oxytocin, 0.54), *F*_(1,45)_ = 4.286, *p* = 0.044, $\eta_{p}^{2}$ = 0.087). That is, compared to placebo, oxytocin produced a general shift of the response bias towards more liberal responding (turning, in absolute terms, a conservative bias value of B_r_ < 0.5 into a liberal bias value of B_r_ > 0.5), but actual recognition memory accuracy (P_r_) was not affected by treatment *per se* or in combination with social context at encoding. The main effects of Pharmacological Treatment on all memory parameters are summarized in Figure 2.

**Figure 3:**
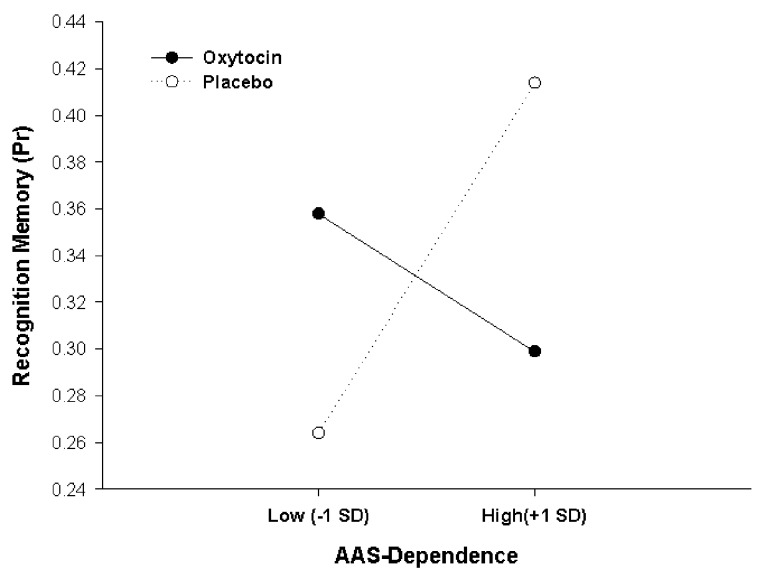
Oxytocin effects on actual memory accuracy (P_r_), depending on adult attachment style (AAS) Dependence. A crossover interaction shows that oxytocin had opposite effects on memory encoding for low and high scorers. Data points selected for illustration refer to 1 standard deviation below and 1 standard deviation above the sample mean.

We then examined whether oxytocin effects on memory accuracy would be the moderated by individual attachment style, entering AAS scores as an additional continuous factor (covariate) in the analysis on P_r_, in addition to the three factors Treatment, Social Context and Word Category from the previous analysis. Because AAS comprises scores on three different facets of attachment style (Dependence, Closeness and Anxiety), three separate analyses were performed for the three subscales, and the significance level for interactive effects was adjusted accordingly (to *p* = 0.05 * 1/3 = 0.017). These analyses of covariances (ANCOVAs) showed that oxytocin effects were critically moderated by participants’ AAS score in Dependence (*F*_(1,43)_ = 8.421, *p* = 0.006, $\eta_{p}^{2}$ = 0.164, for Pharmacological Treatment × Dependence interaction). As illustrated in Figure 3, this interaction results from opposite effects of oxytocin in participants scoring low vs. high on Dependence. For high Dependence scorers (people comfortable with depending on others), exemplified in Figure 3 by participants scoring one standard deviation above the mean, oxytocin had a detrimental effect on memory performance in comparison to placebo (Placebo, P_r_ = 0.414; Oxytocin, P_r_ = 0.299, *p* = 0.04). In contrast, showing a classical crossover interaction, oxytocin exerted a beneficial effect on memory performance for low scorers (people who are wary of depending on others), exemplified in Figure 3 by participants scoring one standard deviation below the mean (Placebo, P_r_ = 0.264; Oxytocin, P_r_ = 0.358, *p* = 0.07). Simple slopes analysis confirmed this pattern of the crossover interaction, showing that a significantly positive slope in the placebo condition (*β* = 0.46, *t*_(22)_ = 2.35, *p* = 0.029) was not only reduced by oxytocin but actually even reversed and turned into a negative slope, although the negative slope under oxytocin did not reach significance *per se* compared to zero (*β* = −0.31, *t*_(23)_ = −1.54, *p* = 0.137). There were no additional higher-order interactions of the Dependence effect with Social Context or Word Category (all *F*s < 1.27, *p*s > 0.26. Scores of AAS Dependence ranged between 13 and 30 (maximally possible range: 6–30) and, critically, did not differ between participants in the oxytocin and the placebo condition (oxytocin: 22.62 ± 4.92, placebo: 24.18 ± 4.20, *t*_(45)_ = 1.15, *p* = 0.26; mean and SD for total sample: 23.40 ± 4.62).

In contrast to Dependence, ANCOVAs performed with the AAS subscales Closeness and Anxiety as covariates showed that these scales did not interact in any way with Pharmacological Treatment alone or in combination with Social Context or Word Category (all *F*s < 2.73, *p*s > 0.10).

### Control Analyses

The same statistical pattern of results as in the main analysis was obtained when, as an alternative method, memory accuracy was derived from signal detection theory as the parameter *d*’ and used as the dependent variable instead of P_r_ (see Snodgrass and Corwin, 1988). Also, a control analysis of the moderation effects, where AAS sores were not treated as a continuous factor but were instead dichotomized by median split, yielded the same statistical results. Furthermore, in another control analysis, we tested for possible order effects by introducing order of social context conditions as an additional independent factor. This control analysis showed that order did not influence the effects of any other factor, including pharmacological treatment (all *F*s < 2.20, *p*s > 0.14).

An additional analysis was performed on the relative distribution of “Remember” and “Know” answers within “old” responses. For this purpose, we calculated the proportion of “Remember” and “Know” answers separately for correct “old” answers (hits) and for wrong “old” answers (false alarms). For both correct and wrong “old” answers, oxytocin did not significantly affect the differential distribution of the two types of answers overall or in interaction with other factors (all *F*s < 2.91, *p*s > 0.05). There was only a significant main effect of Word Category for both correct and wrong “old” answers, indicating that the proportion of “Remember” answers within the “old” answers was generally higher in the “Self” category than in the other two word categories (hits: Self, 0.52, Other, 0.36, None, 0.34, *p* < 0.001 for main effect of Word Category; false alarms: Self, 0.25, Other, 0.14, None, 0.18, *p* < 0.05 for main effect of Word Category).

Finally, we also tested for possible confounding effects of oxytocin-induced unspecific mood changes. PANAS scores for positive and negative mood obtained immediately before and after the encoding task and the retrieval task were averaged as an estimate of positive mood and negative mood during respective task performance. A 2 Pharmacological Treatment (oxytocin, placebo) × 2 Session (encoding, retrieval) ANOVA was performed separately for positive mood and negative mood, with Pharmacological Treatment as a between-subjects factor and Session as a within-subjects factor. Oxytocin did not affect positive or negative mood during task performance overall or in a specific session (all *F*s < 1.63, *p*s > 0.20).

## Discussion

The present study investigated the role of oxytocin in human memory encoding, taking into account both social encoding context and social personality factors, specifically individual attachment style. To our knowledge, this is the first study to apply an interactionist approach to oxytocin effects on memory encoding. According to this interactionist account, oxytocin effects on social cognition and behavior typically do not emerge unconditionally, but dependent on context and/or personality factors (Bartz et al., 2011; Olff et al., 2013; Di Simplicio and Harmer, 2016). The present findings on memory are consistent with this view: Oxytocin (vs. placebo) administered before word encoding had no main effect on memory accuracy in a subsequent recognition memory test. Rather, the effects of oxytocin depended on participants’ attachment style, specifically their social dependence on and trust in others, as assessed by the Dependence subscale of the AAS (Collins and Read, 1990). Specifically, oxytocin enhanced memory accuracy for participants scoring low on Dependence (people who feel uncomfortable depending on others) but reduced memory accuracy for participants scoring high on Dependence (people who feel comfortable depending on others). However, there was no evidence that social context moderated oxytocin effects on memory or that it interacted with any of the three attachment style scales.

Attachment style has been identified as an important personality moderator of oxytocin effects in humans (Bartz et al., 2010a,b, 2015; Olff et al., 2013; Fang et al., 2014; Waller et al., 2015). Our study confirms this specifically with regard to the effect of oxytocin on encoding new material into memory. Regarding the pattern of moderation, most of the previous studies suggest that oxytocin exerts stronger beneficial effects for the less proficient individuals than for the more proficient individuals, who typically do not benefit (Bartz et al., 2011). For example, in a task assessing empathic accuracy, only participants who showed general difficulties in performing the task benefited from oxytocin treatment (Bartz et al., 2010a). Our present study confirms this moderation pattern for the domain of memory encoding, where we found signs of positive oxytocin effects also only for the less proficient individuals, as represented by the participants scoring low in Dependence. Without oxytocin, these participants showed worse memory performance than participants scoring high on Dependence, but this pattern was actually reversed with oxytocin treatment. That is, effects of oxytocin that tended to be beneficial for low scorers were even accompanied by detrimental effects for high scorers.

The association of attachment style with general memory capabilities in the absence of a pharmacologically active drug is a remarkable finding that deserves further scientific attention in the future. Studies on basic cognitive capabilities in relation to social traits are rare, but some findings point to an association between general memory encoding capabilities and individual levels of perspective taking (Stiller and Dunbar, 2007; Wagner et al., 2015). Based on these findings, it is possible that a dependent attachment style is accompanied by stronger perspective taking, and in this way by better overall memory performance. More research is needed to explore these associations in detail.

In contrast to attachment style, social context at encoding had no moderating influence on oxytocin effects *per se* or in combination with attachment style. This was the case despite the fact that we chose a naturalistic manipulation to define the sociality of the context, i.e., interacting (vs. not interacting) with a real other person. However, while this manipulation has a clear advantage regarding ecological validity in comparison to stimulus-based definitions of sociality (Risko et al., 2012; Schilbach et al., 2013), it could have been too weak in the present study to be sufficiently effective. This is because, although our procedure indeed included real social interaction, we also prevented direct perceptual contact between the two interaction partners during the actual phase of task performance. This was done in order to exclude any effects on memory encoding that would be attributable to partner-generated perceptual cues rather than from the sociality vs. non-sociality of the situation *per se*. Our task here used feedback information on the computer screen that reminded participants about whether or not the partner in the adjacent room was simultaneously involved in the current task block. Compared to conditions with full perceptual access to the partner, such a setting increases psychological distance and thus attenuates socially guided effects on memory formation (Wagner et al., 2017). Therefore, this manipulation may have been too subtle in the present context to have substantially influenced oxytocin effects.

Another possibly attenuating factor is that we manipulated the social context in a within-subjects design. That is, the experimental situation as a whole (even in the condition of individual task performance) can be perceived as social because participants knew that another person was participating in the same experiment. This general social character of the experimental session could have made it difficult to create additional differences in the perception of contextual sociality even when implementing the specific task-related social context. Hence, the lack of moderating effects of social context found in the present study does not necessarily mean that such moderating effects would also be absent in manipulations that more strongly highlight the social context. Future studies should examine this possibility, while still controlling for confounds by factors that would allow for non-social explanations of the effects, such as partner-generated perceptual cues that are not present in a non-social context.

It is interesting to compare our findings with those from the only other study that has, so far, applied an interactionist approach to assess how oxytocin affects mnemonic functions (Bartz et al., 2010b). Although we addressed memory encoding while Bartz et al. (2010b) addressed memory retrieval, there are at least two remarkable parallels. First, both studies found that individual attachment style was a critical moderator of oxytocin effects, even when two very different memory functions were investigated. Second, in both studies the moderation expressed itself as a crossover interaction, i.e., oxytocin exerted opposing effects for high vs. low scorers on a certain aspect of attachment style. However, in the study by Bartz et al. (2010b), the critical moderating dimension of attachment style was attachment anxiety, not attachment dependence. Specifically, memory retrieval in their study was negatively affected by oxytocin for high scorers on attachment anxiety, but negatively affected by oxytocin for low scorers on this scale. One might speculate why the two different aspects of individual attachment style are differently involved in oxytocin-dependent mechanisms of memory encoding vs. memory retrieval. Retrieval is a past-oriented process, while encoding is future-oriented. Attachment anxiety implies a (negatively connotated) social focus on the past, while attachment dependence, i.e., acceptance of social interdependence on and trust in others, implies a (positively connotated) social focus on the present and future. Hence, attachment anxiety may be more potent as a moderator of retrieval processes, but attachment dependence may be more potent as a moderator of encoding processes.

However, to highlight another difference, memory retrieval in the study by Bartz et al. (2010b) did not refer to episodic retrieval of experimenter-defined learning material previously presented under experimental control. Rather, it referred to a very specific type of retrieval, i.e., autobiographic retrieval of maternal care and closeness during one’s own childhood. Also, Bartz et al. (2010b) used different assessment instruments than we used in the present study. Specifically, they administered the Experience in Close Relationships scale (ECR) from Brennan et al. (1998), which only distinguishes the two attachment style dimensions of anxiety and avoidance, such that the specific aspects of dependence and closeness (that are basically summarized in the avoidance scale) could not be distinguished. Therefore, some of the differences between the two studies may be simply attributable to procedural differences. Hence, more general conclusions about how different aspects of attachment style moderate oxytocin’s effects on memory encoding vs. memory retrieval must await further investigation.

In most of the previous oxytocin studies using experimental memory paradigms, it is difficult to disentangle oxytocin’s effects on encoding from those on retrieval. This is because these studies have typically been performed in only one single experimental session, in which oxytocin is administered at the beginning and then an experimental memory task (encompassing both encoding and retrieval testing; see above) is performed at the end of the session. However, with this procedure, oxytocin is active in the central nervous system both during encoding and retrieval. Still other studies, such as the one by Bartz et al. (2010b) described in more detail above, were explicitly interested only in the aspect of memory retrieval but not encoding. These studies administered oxytocin only before information is recalled from memory (Bartz et al., 2010b; Cardoso et al., 2016; Liu et al., 2017, Exp.3). Here, we were specifically interested in the influence of oxytocin on memory encoding. Therefore, we made sure that oxytocin treatment could indeed be acutely pharmacologically effective only in the phase of memory encoding but not at memory retrieval by delaying memory testing to a separate session on the day after treatment.

To our knowledge, such a procedure to exclude direct treatment effects on retrieval has so far only been applied in three previous studies, all of which specifically addressed memory encoding for faces (Guastella et al., 2008; Rimmele et al., 2009; Herzmann et al., 2012). Furthermore, only two of these studies directly compared oxytocin effects on encoding of facial (social) and non-facial (non-social) stimuli, with diverging results. Rimmele et al. (2009) reported positive effects of oxytocin on memory encoding of faces but not of non-facial stimuli, but Herzmann et al. (2012) could not replicate this. The inconsistency of these results, even within the studies that ensured oxytocin effects specifically on encoding, suggests that other than stimulus-dependent social factors might determine oxytocin effects on memory encoding. Therefore, we examined here, for the first time, if social context or social personality factors could moderate the effects of oxytocin on memory encoding. However, because we used neutral verbal learning material, it still remains to be determined whether or not the critical moderating role of attachment style similarly holds for non-verbal stimuli or for (verbal or non-verbal) stimuli varying in social or emotional content. In any case, the important methodological necessity to separate effects on encoding vs. retrieval needs to be kept in mind in all future investigations.

Our study also revealed oxytocin effects on memory parameters that emerged regardless of social context or attachment style. Specifically, oxytocin induced participants to respond more liberally in the recognition test, i.e., to accept any word shown in the recognition test as previously seen at encoding. This more liberal responding became directly evident by an increase of the response bias parameter B_r_, but also by an increased FAR and a trend towards a higher HR. Interestingly, this outcome of an oxytocin-induced shift in response bias directly replicates a recent finding that Bate et al. (2015) reported for a study using facial stimuli. However, in contrast to that previous study, in which oxytocin was pharmacologically active during both encoding and retrieval (because both phases took place in the same experimental session after oxytocin vs. placebo treatment), our design ensured that the effect is attributable to oxytocin effects on encoding. That is, oxytocin could not simply cause the effect by directly influencing response behavior at retrieval. Rather, it must have influenced brain processes at encoding in such a way that at later retrieval, any item shown in the recognition test is more likely to induce at least a certain sense of previous episodic encounter with that item than without previous oxytocin treatment. In this respect, the effect must be regarded not simply as a response bias but as an actual memory phenomenon.

We suspect that this effect may be attributable to a specific characteristic of our material. In particular, there were only three semantic categories to which not only all encoded items belonged, but also all distractors in the recognition test. Moreover, learned items and distractors were matched not only on the level of word sets, but even on the level of single items. For example, for each bird in the learning list there was also another bird as a concomitant distractor in the recognition test. Recent findings suggest that one cognitive effect of oxytocin is the facilitation of associative and creative processes (De Dreu et al., 2014). Thus, during encoding, oxytocin might induce a stronger spreading of activation through the semantic network associated with a presented item than under placebo conditions. Accordingly, semantically associated items would be activated together with an actually shown item and thus would be encoded together with it. Because of the semantic constraints of items in our memory test, it is likely that some items co-activated by spreading activation at encoding were shown as distractors at retrieval testing and then falsely judged as previously seen. At the same time, the actually learned items would have been additionally encoded by spreading activation induced by other learned items, such that the HR also increased to some extent because of oxytocin. Future research should test this explanation by using distractors that have systematically varying strength of semantic association in relation to the actually learned material.

In sum, the present study applies, for the first time, an interactionist approach to oxytocin effects (Bartz et al., 2011), specifically to oxytocin’s effects on memory encoding. Consistent with this approach, we found that the effects of oxytocin on memory encoding were moderated by individual personality, specifically attachment dependence, but the effects were not found to be moderated by social context. Further research should be performed to examine social context manipulations that are stronger than the ones employed here, and future studies should continue to explore the oxytocin-induced effect on delayed response bias that we observed here independent of social context or personality. Moreover, more research is needed to better understand which specific biological dispositions linked to attachment style (e.g., genetic variations and differences in central nervous receptor expression) affect memory processes via oxytocinergic mechanisms.

## Author Contributions

UW and GE designed the study; wrote the manuscript. UW performed the experiment and analyzed the data.

## Conflict of Interest Statement

The authors declare that the research was conducted in the absence of any commercial or financial relationships that could be construed as a potential conflict of interest.
